# Development of Real‐Time RT‐PCR Assays for Detection and Typing of Epizootic Haemorrhagic Disease Virus

**DOI:** 10.1111/tbed.12477

**Published:** 2016-02-17

**Authors:** N. S. Maan, S. Maan, A. C. Potgieter, I. M. Wright, M. Belaganahalli, P. P. C. Mertens

**Affiliations:** ^1^ The Pirbright Institute Woking Surrey UK; ^2^ Deltamune Pty Ltd Lyttelton Centurion South Africa; ^3^ Department of Biochemistry Centre for Human Metabolomics North‐West University Potchefstroom South Africa; ^4^ Present address: College of Veterinary Sciences LLR University of Veterinary and Animal Sciences Haryana India

**Keywords:** epizootic haemorrhagic disease (EHD), epizootic haemorrhagic disease virus (EHDV), *Orbivirus*, real‐time RT‐PCR, qRT‐PCR assays, serotype‐specific assays, serogroup‐specific (pan‐reactive) assays, Orbivirus reference collection, *Culicoides*

## Abstract

Epizootic haemorrhagic disease virus (EHDV) is an emerging arboviral pathogen of wild and domestic ruminants worldwide. It is closely related to bluetongue virus (BTV) and is transmitted by adult females of competent *Culicoides* vector species. The EHDV genome consists of ten linear double‐stranded (ds)RNA segments, encoding five non‐structural and seven structural proteins. Genome‐segment reassortment contributes to a high level of genetic variation in individual virus strains, particularly in the areas where multiple and distinct virus lineages co‐circulate. In spite of the relatively close relationship between BTV and EHDV herd‐immunity to BTV does not appear to protect against the introduction and infection of animals by EHDV. Although EHDV can cause up to 80% morbidity in affected animals, vaccination with the homologous EHDV serotype is protective. Outer‐capsid protein VP2, encoded by Seg‐2, is the most variable of the EHDV proteins and determines both the specificity of reactions with neutralizing antibodies and consequently the identity of the eight EHDV serotypes. In contrast, VP6 (the viral helicase), encoded by Seg‐9, is highly conserved, representing a virus species/serogroup‐specific antigen. We report the development and evaluation of quantitative (q)RT‐PCR assays targeting EHDV Seg‐9 that can detect all EHDV strains (regardless of geographic origin/topotype/serotype), as well as type‐specific assays targeting Seg‐2 of the eight EHDV serotypes. The assays were evaluated using orbivirus isolates from the ‘Orbivirus reference collection’ (ORC) at The Pirbright Institute and were shown to be EHDV pan‐reactive or type‐specific. They can be used for rapid, sensitive and reliable detection and identification (typing) of EHDV RNA from infected blood, tissue samples, homogenized *Culicoides*, or tissue culture supernatant. None of the assays detected RNA from closely related but heterologous orbiviruses, or from uninfected host animals or cell cultures. The techniques presented could be used for both surveillance and vaccine matching (serotype identification) as part of control strategies for incursions in wild and domestic animal species.

## Introduction

Epizootic haemorrhagic disease virus (EHDV) is an emerging arboviral pathogen of wild and domestic ruminants worldwide. It is closely related to bluetongue virus (BTV) and is transmitted by adult females of competent *Culicoides* vector species. EHDV was formally identified by Shope and co‐workers (Shope et al., 1955), who isolated the virus from white‐tailed deer (WTD) (*Odocoileus virgininianus*) in New Jersey, and it is regarded as the most important infectious disease of WTD in the USA (Nettles, 1992).

Although all ruminants appear to be susceptible to EHDV infection, the clinical disease varies considera‐bly with the host species. In general, domestic ruminants (cattle, sheep and goats) infected with EHDV tend to develop inapparent‐to‐mild infections (MacLachlan and Osburn, 2004; Allison et al., 2010, 2012; Savini et al., 2011). Initially, only Ibaraki virus (a strain of EHDV‐2 from Japan) was known to cause disease in cattle (Kitano, 2004; Savini et al., 2011). However, in recent years, the association of EHDV with morbidity and mortality in cattle has increased dramatically in many parts of the world. EHDV‐7 was recently involved in outbreaks in Israeli cattle, although with low (<1%) mortality rates but high (5–80%) morbidity rates (Yadin et al., 2008). EHDV outbreaks were also reported in cattle from Réunion Island in 2003 (Breard et al., 2004); Algeria and Morocco in 2006 (reported as EHDV‐318/EHDV‐9; reclassified as EHDV‐6) (Yadin et al., 2008; Anthony et al., 2009a); western Turkey in 2007 (EHDV‐6) (Temizel et al., 2009); French Guiana in 2012 (Viarouge et al., 2014); and Tunisia during October 2015 (ProMED‐mail (2015) Epizootic hemorrhagic disease) suggesting that the geographical distribution and/or the clinical disease associated with EHDV infection is increasing.

It is unclear whether these changes are caused by an increased susceptibility of the host populations, changes in local vector competency or activity, changes in the environmental conditions or ecosystems, increased virus movement and spread, or the introduction and emergence of novel EHDV strains, possibly with increased pathogenicity. The extended host range of EHDV‐6 and 7 in cattle has caused major concerns for international authorities, and in 2008, the disease was included in the OIE list of multispecies/transboundary diseases (OIE, 2014).

Although closely related and showing low‐level serological cross‐reactions with bluetongue virus (BTV – the prototype orbivirus)*, ‘Epizootic haemorrhagic disease virus’* represents a distinct species within the genus *Orbivirus,* family *Reoviridae*. Like BTV, EHDV has a wide distribution around the world, and there is clear evidence for variations in the virus genome‐sequences that correlate with the geographic origin of the virus (topotypes). There is even a size difference in EHDV genome segment 9 between eastern and western topotype viruses (Anthony et al., 2009a, 2010, 2011). Despite a close relationship between BTV and EHDV, pre‐existing immunity to BTV in a region is not expected to give any significant protection against the introduction and infection by EHDV (Eschbaumer et al., 2012). In fact, several authors have reported the co‐circulation of EHDV and BTV: in French Guiana; Reunion Island; Kenya; and the USA (Sailleau et al., 2012; Toye et al., 2013; Cetre‐Sossah et al., 2014; Viarouge et al., 2014).

Like BTV, EHDV has a genome composed of ten segments of linear double‐stranded RNA identified as genome segment 1–10 (Seg‐1 to Seg‐10) in order of decreasing molecular weight, encoding a total of five non‐structural and seven structural proteins (Mertens et al., 2005; Attoui et al., 2012; Stewart et al., 2015). The reassortment (exchange) of orbivirus genome segment between different strains belonging to the same virus species can generate new ‘virus genotypes’ contributing to a high level of genetic variation in the progeny virus strains (Nomikou et al., 2015).

Epizootic haemorrhagic disease virus proteins ‘VP2’ and ‘VP5’ (also designated as outer‐capsid protein 1 and 2 [OC1 & OC2]) form the outer layer of the virus particle and are encoded by Seg‐2 and Seg‐5, respectively. Both VP2(OC1) and VP5(OC2) vary in a serotype‐specific manner and are the least conserved of the EHDV proteins/genome segments, respectively (Maan et al., 2007a, 2011; Anthony et al., 2009b). VP2(OC1) of EHDV and BTV is responsible for receptor binding on the host‐cell and determines virus serotype through the specificity of its interactions with neutralizing antibodies (Huismans et al., 1979). VP5(OC2) has been shown to enhance the neutralizing antibody response induced by VP2(OC1) alone (Patel and Roy, 2014). In contrast, the viral helicase VP6(Hel) (encoded by Seg‐9) is a minor structural component of virus core, and it is one of the most conserved of the orbivirus proteins, showing sequence variations that correlate with the virus serogroup/species and with the geographic origins of the virus isolate (topotype) (Mertens and Diprose, 2004; Mertens et al., 2004).

According to earlier classifications, ten serotypes/strains of EHDV were recognized: serotypes 1–8, EHDV‐318 and Ibaraki virus (IBAV) (Mertens et al., 2005). However, genetic and phylogenetic analyses of the outer‐capsid proteins responsible for serotype specificity [VP2(OC1) and VP5(OC2)] have led to a proposal to condense EHDV classification into only seven serotypes: with the inclusion of EHDV‐3 into EHDV‐1; EHDV‐318 into EHDV‐6; IBAV into EHDV‐2 (Anthony et al., 2009b; Maan et al., 2010). More recently, there is evidence for a novel eighth serotype identified as EHDV‐9 (to avoid confusion with earlier strains listed as EHDV‐1 to EHDV‐8), isolated from an alpaca in South Africa (Wright, 2013).

Initially, EHDV was isolated in embryonated chicken eggs (ECE), and/or adaptation to cell culture. Members of the EHDV serogroup/virus species could be identified by serological methods, including ELISA tests, for both antigen and antibody detection (Afshar et al., 1992; Thevasagayam et al., 1995, 1996). EHDV could also be ‘serotyped’ using either ‘virus neutralization tests’ (VNT) or ‘serum neutralization tests’ (SNT) (Pearson et al., 1985; Howell et al., 2002; Maan et al., 2010; OIE, 2014). However, these serological methods are labour intensive, time consuming (weeks) and require access to well‐characterized ‘reference’ virus strains and/or antisera. They also have low sensitivity and specificity, particularly in areas where multiple virus serotypes are co‐circulating, leading to mixed virus isolates, cross‐reactions and mis‐identification or a failure to identify individual serotypes (Maan et al., 2010).

So far there has been limited interest in developing vaccines to control the EHD or EHDV circulation. To date, an autogenous vaccine that can be used only in captive wild deer has been developed in the USA (OIE, 2014). In Japan, both live modified and inactivated vaccines have been developed to control Ibaraki disease in cattle (Ohashi et al., 1999). These studies with EHDV and with other related orbiviruses indicate that vaccines are likely to be serotype‐specific. The rapid and reliable detection and identification of different EHDV types is, therefore, likely to be an important component of any surveillance/vaccination/control strategy.

Detection of EHDV has previously been reported by molecular assays, using virus species/serogroup‐specific (pan‐reactive) primers and probes targeting conserved genome segments 5, 7 and/or 10 (NS1, VP7 and/or NS3 genes, respectively) (Aradaib et al., 1995, 2003; Wilson et al., 2009a,b; Viarouge et al., 2015). RT‐PCR‐based conventional molecular assays have also been developed to identify individual EHDV types, more rapidly and more reliably than by conventional serological methods (Maan et al., 2010).

This article describes the development and evaluation of a quantitative (q)RT‐PCR to amplify Seg‐9, making it possible to detect any EHDV strain regardless of serotype or topotype. A set of eight individual (q)RT‐PCR assays were designed targeting Seg‐2, to detect and identify each of the different EHDV serotypes. Assay specificity was evaluated using a wide range of EHDV isolates from the Orbivirus reference collection (ORC), including reference, vaccine and field strains involved in recent disease outbreaks in the Mediterranean region and North America (Yadin et al., 2008; Allison et al., 2010; Kedmi et al., 2010).

## Material and Methods

### Primers and probe design

The design of primers and probes for the detection and typing of EHDV was based on Seg‐9 and Seg‐2 nucleotide sequences of EHDV reference strains generated during this study, using the previously described method (Maan et al., 2007b) combined with nucleotide sequences available in the public domain (GenBank) (Table 1).

**Table 1:** Sequence data used to design real‐time RT‐PCR assays

Virus serotype	Origin	ORC reference number^b^	Seg‐9 Acc. number	Seg‐2 Acc. number
**Reference strains** a				
EHDV‐1e	Australia	AUS1995/02	–	HM156728
EHDV‐1w	USA	USA1955/01	AM744985	AM744978
EHDV‐1w	Nigeria	NIG1967/01	AM745015	AM745008
EHDV‐2w	Canada	CAN1962/01	AM745005	AM744998
EHDV‐2e	Japan	JAP1959/01	AM745085	AM745078
EHDV‐2e	Australia	AUS1979/05	AM744995	AM744988
EHDV‐4w	Nigeria	NIG1968/01	AM745025	AM745018
EHDV‐5e	Australia	AUS1977/01	AM745035	AM745028
EHDV‐6e	Australia	AUS1981/07	AM745045	AM745038
EHDV‐6w	Bahrain	BAR1983/01	AM745075	AM745068
EHDV‐7e	Australia	AUS1981/06	AM745055	AM745048
EHDV‐7w	Israel	ISR2006/013	–	HM156731
EHDV‐8e	Australia	AUS1982/06	AM745065	AM745058
EHDV‐9w	RSA	Not available	–	–

a Set of eight monotypic EHDV reference strains (Anthony et al., 2009b; Wright, 2013).

b Orbivirus reference collection (ORC), The Pirbright Institute.

Sequences for Seg‐2 or Seg‐9 were aligned and analysed using MEGA v. 5 (Tamura et al., 2011). Conserved regions in Seg‐9 were identified across all eight serotypes, as targets for the design of primers and probes, for use in pan‐reactive assays, in accordance with TaqMan specifications (Table 2).

**Table 2:** List of primers and probes for EHDV pan‐reactive and type‐specific assays

Assay type	Oligo name	Oligo sequence (5′–3′)
Seg‐9‐based/group‐specific		
	EHDV/Seg‐9/F/15‐32	ATGTCAGCTGCGGTYTTG
	EHDV/Seg‐9/R/112‐85	TCCCAATCAACTAARTGRATYTGVATCT
	EHDV/Seg‐9/P/69‐48	CCTCGGTCGAACGTTGGATCAC
Seg‐2‐based/type‐specific		
	EHDV‐1w/Seg‐2/246‐275F	GAATAATTCGYTAYGAGAATAAARCYAAAG
	EHDV‐1w/Seg‐2/364‐341R	TCTATGYGYCTCRTCCATTCTYGG
	EHDV‐1wSeg‐2/305‐329P	CAGCTGCGGTCATCTATTAGGCATC

	EHDV‐1e/Seg‐2/260‐278F	GACAGCAAAAGTAGAGGAG
	EHDV‐1e/Seg‐2/359‐341R	GGGTTTCATCCATTTTTGG
	EHDV‐1e/Seg‐2/304‐329P	CGAGTTACGATCATCTATCAGGCATC

	EHDV‐2e/Seg‐2/2540‐2559F	GAAGTATTGGTTAAATATCG
	EHDV‐2e/Seg‐2/2627‐2606R	GCTRAAATGCGTATTCAATGG
	EHDV‐2e/Seg‐2/2564‐2590P	CCCACGCAGGGAGATCACCGAYTYAAC

	EHDV‐2w/Seg‐2/1910‐1934F	TATGTTAAATGTATTGAATTATACG
	EHDV‐2w/Seg‐2/2087‐2069R	TCTCATCCCGACCAACACT
	EHDV‐2w/Seg‐2/2027‐1997P	CTCTTCATCCGGATCCTGATATACCTCCATC

	EHDV‐4w/Seg‐2/339‐360F	GGACTTTGAATCATTGATGTTG
	EHDV‐4wSeg‐2/445‐426R	GCACGTCAGTTTGCTGCAGT
	EHDV‐4w/Seg‐2/415‐387P	CTCGCCGCCCTGTGAAGTCAACTCCTGCC

	EHDV‐5w/Seg‐2/783‐798F	TGGTGAGCGTGGTGCG
	EHDV‐5w/Seg‐2/863‐839R	GCAGCTATATCATCTAAAGCAATTG
	EHDV‐5w/Seg‐2/809‐830P	CACGCGAAGGAATAGCCCCATC

	EHDV‐6e/Seg‐2/601‐622F	GATTGTAATAGGAGAGATTAAG
	EHDV‐6e/Seg‐2/743‐724R	GACCCAAAGCCGCCTGGATT
	EHDV‐6e/Seg‐2/638‐669P	CGTCAAAATGTCATAACTCGGCAGATGATACC

	EHDV‐7e/Seg‐2/212‐229F	GATGGGCTATGATATCAT
	EHDV‐7e/Seg‐2/323‐304R	TAATCCCTGTTCTTCACCTT
	EHDV‐7e/Seg‐2/280‐301P	CCAAGAACTCAAAGGATCCGGC

	EHDV‐7w/Seg‐2/2134‐2150F	GACAAATTTTGATGCCG
	EHDV‐7w/Seg‐2/2241‐2225R	GAGAAAAGTTGGGCGCT
	EHDV‐7w/Seg‐2/2220‐2194P	CCTCTAAGATCTCATCCCGTCTCTCCC

	EHDV‐8e/Seg‐2/886‐900F	GCGGATCGAAGAAAT
	EHDV‐8e/Seg‐2/1021‐1001R	AGTGGTCTTAACCAAGTTCTG
	EHDV‐8e/Seg‐2/992‐966P	CGTTATACAATCTGACTCGCGCCCCCC

	EHDV‐9w/S2/2147‐2169F	ACTAAATGAAGAAGAGATACGTA
	EHDV‐9w/S2/2251‐2228R	GCTATAATGTTATAGAAATTTGGT
	EHDV‐9w/S2/2192‐2226P	CTAATGCCCGCATTATTGCTTCCCGATGGT

The names of the oligonucleotide primers and probes indicate the group‐ or type‐specificity of the assay. Seg‐9 indicates the group‐specific assays while Seg‐2 indicates respective type‐specific assays. F, R and P stand for ‘forward primer’, ‘reverse primer’ and ‘probe’, respectively. The numbers represent annealing positions on the genome segment.

Genome segment 2 sequences were analysed collectively and separately for each of the eight EHDV serotypes. Full‐length Seg‐2 sequences already obtained for strains of the different EHDV serotypes were compared and unique regions were identified (showing intra‐typic conservation and hetero‐typic variation), as a basis for the design of serotype‐specific RT‐PCR primers and probes (Anthony et al., 2009b). Wherever possible, these analyses included data for multiple isolates of each type, from different geographical origins, to help ensure that the resulting primers and probes would amplify and detect Seg‐2 from different topotypes within the same serotype. The probes were labelled at their 5′ and 3′ ends with 6‐carboxy‐2′,4,4′,5′,7,7′‐hexachlorofluorescein (6‐HEX) and Black Hole Quencher‐1 (BHQ‐1), respectively. All oligonucleotides were synthesized by Eurogentec, UK. Primers were PAGE‐purified and probes were HPLC‐purified.

At least two primer pairs were designed targeting Seg‐2 of either eastern or western topotypes of each EHDV serotype (identified by the letters ‘e’ and ‘w’, respectively; Table 2). Individual primers and probes were named as follows: ‘EHDV’, followed by a number indicating the serotype; then ‘S2’ or ‘S9’ to indicate they target Seg‐2 or Seg‐9; ‘F’ representing forward, or ‘R’ representing reverse primers; ‘P’ representing probe; and a number corresponding to the nucleotide binding position of the primer in Seg‐2. The Seg‐9 assay was designed to detect both eastern and western EHDV topotypes.

The sequences of the different primers and probes were evaluated *in silico* to ensure no cross‐reactions with closely related orbiviruses. The relevant genome segments of all 29 species of orbiviruses were used for *in silico* analysis (Maan et al., 2007a; Belaganahalli et al., 2011, 2012; Mertens and Attoui, 2014).

### Virus isolates

A panel of 62 isolates representing seven of the eight known EHDV serotypes, including reference, field and vaccine strains from different geographical locations, was used for the validation of real‐time RT‐PCR assays (Table 3). The EHDV‐9 assay was validated by *in silico* comparisons only.

**Table 3:**
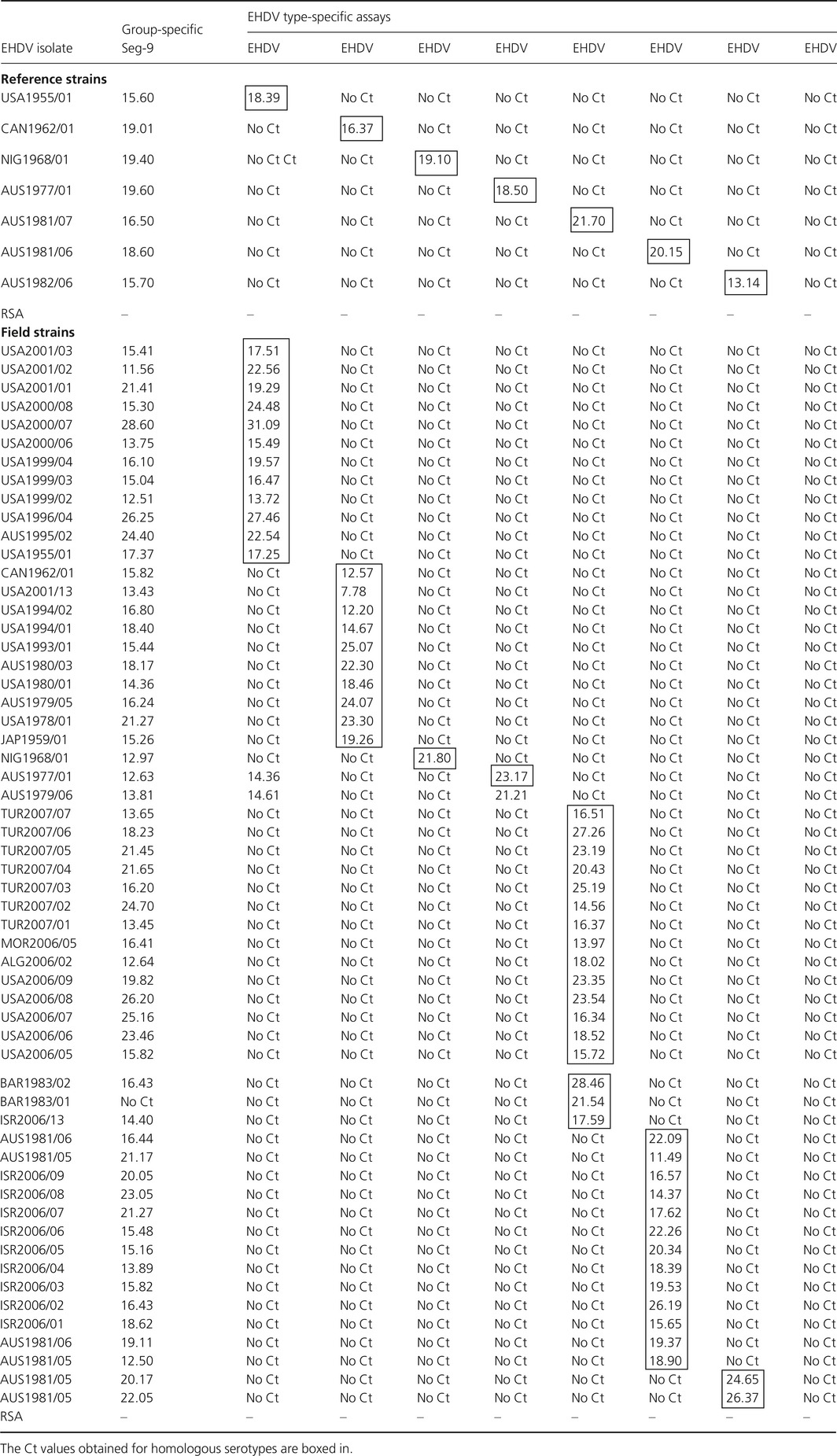
EHDV isolates used to validate pan‐reactive (Seg‐9) and type‐specific (Seg‐2) real‐time RT‐PCR assays

The different EHDV and other orbivirus isolates (listed in Table 2) were isolated in KC cells derived from *Culicoides sonorensis* (Wechsler and McHolland, 1988) and grown further in BHK‐21 clone 13 cell monolayers (European Collection of Animal cell Cultures [ECACC – 84100501]). The infected mammalian cells were harvested when 70–100% cytopathic effect (CPE) was observed, while KC cells were harvested after 7 days. Previously established reference strains of EHDV were used in these studies (Maan et al., 2010).

### RNA sample preparation

Double‐stranded RNA was extracted either from tissue culture supernatant by BioRobot Universal (Qiagen, UK), or from EHDV‐infected cells (using Trizol Reagent^®^; Invitrogen, UK) (Attoui et al., 2000). In the first method, 240 *μ*l of infected tissue culture supernatant was added to 40 *μ*l of protease (Qiagen) and 360 *μ*l of lysis buffer (Roche, UK). Total nucleic acid (50 *μ*l) was extracted from this solution using the BioRobot platform UNIrcV23A V3.0 protocol. Total nucleic acid from uninfected tissue culture supernatants, cattle blood or homogenized *Culicoides* was also extracted using BioRobot Universal (Qiagen).

### Reverse transcription reactions and amplification of Seg‐2 and Seg‐9

Different primers–probe sets were used to amplify a fragment of each of the targeted genome segments, using SuperScript III/Platinum Taq One‐Step qRT‐PCR Kit (Invitrogen). To achieve high efficiency, the individual steps of the RT‐PCR reaction were optimized for each assay, including primers concentrations, primers–probe ratios, magnesium concentration, reverse transcription temperature and period, annealing temperature and period (Table 4). dsRNA samples were heat‐denatured prior to addition to the reaction mix (Shaw et al., 2007). To avoid accidental contamination, the reaction mix was prepared in a ‘clean work station’ and template was added in a separate ‘work station’. Amplification for all of the assays was carried out in Mx3005p (Stratagene, UK) using the same conditions as follows: 55°C for 30 min, 95°C for 10 min followed by 50 cycles of 95°C for 30 s and 60°C for 1 min. Fluorescence was measured during annealing/extension phase of the reaction. Cycle threshold (Ct) values were measured at the point at which the sample fluorescence signal crossed a threshold value (the background level). Negative results (for assays that did not exceed this level of signal) are reported as ‘No Ct’.

**Table 4:** RT‐PCR assay composition

Reagent	Assay detecting segment	
	Seg‐9	Seg‐2
Forward primer (*μ*l)	1 (15 *μ* m)	1 (10 *μ* m)
Reverse primer (*μ*l)	1 (15 *μ* m)	1 (10 *μ* m)
Probe (*μ*l)	0.5 (5 *μ* m)	0.5 (5 *μ* m)
50 mM MgSO4^a^ (*μ*l)	1	1
SuperScript III RT/Platinum *Taq* Mix^a^ (*μ*l)	0.5	0.5
2× Reaction Mix^a^ (*μ*l)	12.5	12.5
Nuclease free water (*μ*l)	4.5	4.5
dsRNA(*μ*l)	4.0	4
Total volume(*μ*l)	25	25

a SuperScript III/Platinum Taq one‐step qRT‐PCR kit (Invitrogen).

### Diagnostic sensitivity and specificity

A panel of EHDV isolates from different geographical locations, including the initial seven known serotypes (Table 3), was tested in triplicate, with the EHDV pan‐reactive assay targeting Seg‐9. The Seg‐2‐based assays (type‐specific assays) were also tested with both homologous and heterologous serotypes to ensure specificity and diagnostic sensitivity. Representative isolates of other *Orbivirus* species, including bluetongue virus (BTV), African horse sickness virus (AHSV), equine encephalosis virus (EEV) and Peruvian horse sickness virus (PHSV) were used to assess diagnostic specificity of Seg‐9‐based assays as listed in Table 5. To ensure that there were no false positives caused by cross‐reactions with the host species, total nucleic acid extracted from uninfected BHK and KC cell culture supernatants, cattle blood and individually homogenized *C. sonorensis* were also tested with uniformly negative results (Table 5).

**Table 5:** Specificity of EHDV group‐specific (Seg‐9) and type specific (Seg‐2) assays

Virus species‐serotype	Origin	ORC reference number	Ct values	
			Seg‐9	Seg‐2
EHDV‐1w	USA	USA1955/01	15.60	18.39
EHDV‐1w	Nigeria	NIG1967/01	16.79	19.32
EHDV‐2w	Canada	CAN1962/01	19.01	16.37
EHDV‐2e	Japan	JAP1959/01	19.34	17.66
EHDV‐2e	Australia	AUS1979/05	21.10	17.72
EHDV‐4w	Nigeria	NIG1968/01	19.40	19.10
EHDV‐5e	Australia	AUS1977/01	19.60	18.50
EHDV‐6e	Australia	AUS1981/07	16.50	21.70
EHDV‐6w	Bahrain	BAR1983/01	18.23	22.41
EHDV‐7e	Australia	AUS1981/06	18.60	20.15
EHDV‐7w	Israel	ISR2006/013	16.49	21.03
EHDV‐8e	Australia	AUS1982/06	15.70	13.14
EHDV‐9w	RSA	–	–	–
AHSV‐1	RSA	RSArah1/03	No Ct	No Ct
AHSV‐2	RSA	RSArah2/03	No Ct	No Ct
AHSV‐3	RSA	RSArah3/03	No Ct	No Ct
AHSV‐4	RSA	SPArah4/03	No Ct	No Ct
AHSV‐5	RSA	RSArah5/03	No Ct	No Ct
AHSV‐6	RSA	RSArah6/03	No Ct	No Ct
AHSV‐7	Kenya	KENrah7/03	No Ct	No Ct
AHSV‐8	RSA	RSArah8/03	No Ct	No Ct
AHSV‐9	Pakistan	PAKrah9/03	No Ct	No Ct
EEV‐1	RSA	RSA1967/03	No Ct	No Ct
EEV‐2	RSA	RSA1971/06	No Ct	No Ct
EEV‐3	RSA	RSA1974/06	No Ct	No Ct
EEV‐4	RSA	RSA1976/03	No Ct	No Ct
EEV‐5	RSA	RSA1976/06	No Ct	No Ct
EEV‐6	RSA	RSA1991/03	No Ct	No Ct
PHSV	Peru	PER1997/01	No Ct	No Ct
BTV‐1	RSA	RSArrrr/01	No Ct	No Ct
BTV‐2	RSA	RSArrrr/02	No Ct	No Ct
BTV‐3	RSA	RSArrrr/03	No Ct	No Ct
BTV‐4	RSA	RSArrrr/04	No Ct	No Ct
BTV‐5	RSA	RSArrrr/05	No Ct	No Ct
BTV‐6	RSA	RSArrrr/06	No Ct	No Ct
BTV‐7	RSA	RSArrrr/07	No Ct	No Ct
BTV‐8	RSA	RSArrrr/08	No Ct	No Ct
BTV‐9	RSA	RSArrrr/09	No Ct	No Ct
BTV‐10	RSA	RSArrrr/10	No Ct	No Ct
BTV‐11	RSA	RSArrrr/11	No Ct	No Ct
BTV‐12	RSA	RSArrrr/12	No Ct	No Ct
BTV‐13	RSA	RSArrrr/13	No Ct	No Ct
BTV‐14	RSA	RSArrrr/14	No Ct	No Ct
BTV‐15	RSA	RSArrrr/15	No Ct	No Ct
BTV‐16	RSA (originally from Pakistan)	RSArrrr/16	No Ct	No Ct
BTV‐17	RSA	RSArrrr/17	No Ct	No Ct
BTV‐18	RSA	RSArrrr/18	No Ct	No Ct
BTV‐19	RSA	RSArrrr/19	No Ct	No Ct
BTV‐20	RSA	RSArrrr/20	No Ct	No Ct
BTV‐21	RSA	RSArrrr/21	No Ct	No Ct
BTV‐22	RSA	RSArrrr/22	No Ct	No Ct
BTV‐23	RSA	RSArrrr/23	No Ct	No Ct
BTV‐24	RSA	RSArrrr/24	No Ct	No Ct
BTV‐25	Switzerland	SWI2008/01	No Ct	No Ct
BTV‐26	Kuwait	KUW2010/02	No Ct	No Ct
BTV‐27	Corsica, France	strain 37	No Ct	No Ct
BTV‐28	Middle‐east	–	No Ct	No Ct
BTV‐29	RSA	–	No Ct	No Ct
*C. sonorensis*			No Ct	No Ct
Uninfected BHK cells			No Ct	No Ct
Uninfected Vero cells			No Ct	No Ct
Uninfected KC cells			No Ct	No Ct
Uninfected cattle blood			No Ct	No Ct

Representatives of different serotypes and topotypes from five different *Orbivirus* species (AHSV, EEV, PHSV, EHDV and BTV) were tested to confirm the specificity of assays for EHDV dsRNA. Further details on these isolates can be obtained from ORC http://www.reoviridae.org/dsRNA_virus_proteins/ReoID/viruses-at-iah.htm. EEV = Equine encephalosis virus; PHSV = Peruvian horse sickness virus; EHDV = Epizootic haemorrhagic disease virus; BTV = Bluetongue virus. RSA = Republic of South Africa.

### Assay sensitivity and efficiency

The analytical sensitivity of the Seg‐9 pan‐reactive assay was assessed using a dilution series of the quantified dsRNA genome of EHDV‐1 (AUS1995/02). Additionally, sensitivity of the Seg‐9 pan‐reactive PCR was also assessed using a recombinant plasmid DNA having a 97‐bp insert (Seg‐9‐specific amplicon). In the type‐specific assays (Seg‐2), analytical sensitivity was assessed using a dilution series of genomic dsRNA of the respective EHDV reference strains (Maan et al., 2010).

The dsRNA standards were prepared as follows: viral dsRNA (of AUS1995/02 or other reference strains) was extracted as previously described (Attoui et al., 2000), assessed for any ssRNA remains in 1% agarose gel electrophoresis, and the concentration of dsRNA was determined with NanoDrop (Thermo Fisher Scientific, USA). The number of dsRNA copies was calculated with the formula: *Y* = (*X*/(*a* × 680)) × 6.022 × 10^23^, where *Y* = molecules/*μ*l; *X* = g/*μ*l dsRNA; *a* = viral genome length in nucleotides; 680 is the average molecular weight per nucleotide of dsRNA.

To test analytical sensitivity, a 10‐fold dilution series of dsRNA (10^11^ to 10^0^ copies per *μ*l) was made in a sample of RNA extracted from uninfected BHK cells supernatant, then tested in triplicate. To test analytical sensitivity of the Seg‐9 pan‐reactive assay using recombinant plasmid DNA, a 10‐fold dilution series of plasmid DNA (10^11^ to 10^0^ copies per *μ*l) was made in TE dilution buffer and then tested in triplicate. The number of plasmid DNA copies was calculated with the formula: *Y* = (*X*/(*a* × 660)) × 6.022 × 10^23^, where *Y* = molecules/*μ*l; *X* = g/*μ*l dsDNA; *a* = plasmid plus insert length in nucleotides; 660 is the average molecular weight per nucleotide of dsDNA.

Viral dsRNA dilution series were used to generate standard curves by linear regression methods, setting Ct values as dependent, and the dsRNA concentrations as independent variables. The slope of the standard curves for the seven reference strains of EHDV in all optimized pan‐reactive‐ and serotype‐specific assays was then used to estimate the efficiency of the individual assays in detection of each of the reference strain. The efficiency was calculated by the formula $E\%=(10^{1/\text{slope}}-1)\times 100$. Efficiencies of Seg‐2 and Seg‐9 assays were also estimated on the basis of standard curves plotting Ct values against corresponding log dsRNA copy number per reaction.

## Results

### Sequence comparisons and assay design

#### Seg‐9‐based pan‐reactive EHDV assay

Comparisons of Seg‐9 nucleotide sequences, from the eight reference strains of EHDV, along with publicly available sequence data (Table 1), identified conserved regions for the development of pan‐reactive qRT‐PCR assays (Table 2). Some redundant bases were introduced within the Seg‐9 forward and reverse primers to accommodate nucleotide differences between individual virus strains to ensure assay sensitivity and specificity (Table 2).

#### Seg‐2‐based assays

Publically available and newly generated nucleotide sequences for Seg‐2 of the eight EHDV serotypes were analysed and compared (Table 1). Regions unique to each virus type were identified allowing type‐specific primers and probes to be designed targeting these regions (Table 2).

### Assay specificity

#### Seg‐9‐based assays

A panel of monotypic EHDV reference strains, representing the seven initial serotypes of EHDV (Table 3), was tested in triplicate with pan‐reactive assays, targeting Seg‐9.

The specificity of the EHDV Seg‐9‐based pan‐reactive assay was further evaluated using 62 different field and vaccine strains of EHDV serotypes 1, 2, 4, 5, 6, 7, 8 and 9, collected from different geographical locations (including different topotypes) (Table 3). All of the isolates produced amplification in this assay although with some Ct value difference. These variations in Ct values suggest minor differences in the amount of ‘target’ in the isolates tested or could be due to minor differences in sensitivity/efficiency of the assay with different strains.

‘No Ct’ results were consistently generated with nucleic acid of uninfected *C. sonorensis*, uninfected cattle blood or cell culture supernatants (KC cells, BHK and Vero cells), or dsRNA from other closely related *Orbivirus* species (AHSV, EEV, PHSV and BTV) (Table 5). *In silico* analysis of Seg‐9 sequence data for 29 *Orbivirus* species (Belaganahalli, 2012) indicated that neither primers nor probes of the EHDV pan‐reactive assay would bind to or amplify their RNA.

#### Seg‐2‐based (type‐specific) assays

The RNAs of the seven monotypic EHDV reference strains were tested with the different ‘typing assays’ (Table 3). In each case, amplification was only observed with the homologous assay. Further testing using field strains of EHDV serotypes 1, 2, 4, 5, 6, 7 and 8, also produced positive signals but only with the homologous serotype. These qRT‐PCR results were confirmed by sequencing and phylogenetic analyses of Seg‐2 for majority of the isolates tested (Table 3).

The North African field isolates of EHDV from 2006 (ALG2006/02 and MOR2006/05) were initially tested positive in EHDV ‘pan‐reactive’ qRT‐PCR, giving cycle threshold (Ct) values of 12 and 14, respectively. These isolates were subsequently also tested by qRT‐PCR, using the ‘type‐specific’ primers and probes listed in Table 2. The results provided the first indication that both isolates belong to the western topotype of EHDV‐6 (EHDV‐6w). No cDNAs were generated with any of the other primer pairs, including those directed against eastern strains of EHDV‐6e.

Similarly, an Israeli isolate of 2006 (ISR2006/13) was found to contain both EHDV‐6w and EHDV‐7w when tested using real‐time RT‐PCR. These results were confirmed using conventional type‐specific RT‐PCR assays as well as sequencing of VP2 gene (Maan et al., 2010). Isolates from the USA during 2006 (USA2006/05 and USA2006/09) also tested positive for EHDV‐6e and were shown to be closely related to strains from Australia (AUS1981/07) in VP2 gene sequencing studies. Several 2007 isolates of EHDV from Turkey (TUR2007/01 to TUR2007/06) contained EHDV‐6w, with VP2 gene sequences closely related to BAR1983/01.

Nucleic acid preparations derived from uninfected hosts (cattle blood and *C. sonorensis*) or uninfected cell culture supernatants (KC cells, C6/36 cells, BHK cells and Vero cells) gave ‘No Ct’ values in any of the seven type‐specific assays.

### Assay sensitivity, efficiency, repeatability and reproducibility

#### Seg‐9‐based (pan‐reactive) assay

Efficiency rates for the Seg‐9 assay was calculated, based on a dilution series of viral RNA purified from EHDV‐1 (AUS1995/02), showing 101% efficiency, estimated over seven (10^1^–10^7^) log dilutions with *R* ^*2*^ exceeding 0.99 (Table S1a).

The assay targeting Seg‐9 consistently detected down to three copies of EHDV‐1e (AUS1995/02), EHDV‐2e (JAP1959/01) or EHDV‐1w (NIG1967/01) RNA per reaction, in three independent repeats, with average Ct values of 36.02 (Table S1b). EHDV‐1 RNA was diluted in RNA extracted from EHDV negative blood and tested in different runs (several days apart) with good repeatability. The different serotypes of EHDV when tested in triplicates (in different runs) show good reproducibility for EHDV detection.

#### Seg‐2‐based assays (serotyping assays)

RNA of the homologous EHDV serotype was detected by seven of the ‘typing’ assays, at all eight dilutions down to 2–139 copies (Table S2). Due to the unavailability of an isolate of EHDV‐9, the assay for this type was only evaluated *in silico*.

Efficiency rates were calculated for the different typing assays, on the basis of dilutions series of dsRNA, giving values between 85.6% and 110.8% (Table S2) reflected by a range of slope values (between −3.1 and −3.7). All seven of the Seg‐2 assays that were tested against their homologous serotypes showed linearity (*R* ^2^ > 0.97) (Table S2). Due to the unavailability of an EHDV‐9 isolate, it was not possible to test the EHDV‐9 assay in this way.

## Discussion

Epizootic haemorrhagic disease virus and related orbiviruses comprise a group of arthropod‐borne RNA viruses that are capable of rapid genotypic and phenotypic change. Considerable variation can occur in the severity and types of disease manifestations associated with orbivirus infections. Host and viral factors, as well as environmental circumstances under which the virus and host interact, can all influence the outcome of infection. In North America, white‐tailed deer are most severely affected, and although black‐tailed deer (mule deer) are also susceptible to infection their survival rate is much higher. Pronghorn antelope and elk are only mildly affected (Allison et al., 2010). Although EHDV has long been known to cause occasional disease outbreaks in cattle, several outbreaks have been reported since 2005, in five Mediterranean countries as well as in the USA, showing clinical signs that are very similar to those of bluetongue in sheep, although the latter is usually asymptomatic in calves (Breard et al., 2013; Cetre‐Sossah et al., 2014).

Here, we report the development of TaqMan real‐time RT‐PCR assays for detection and typing of EHDV RNA in diagnostic samples or infected tissue cultures, from the complete range of eight known serotypes. The highly conserved Seg‐9 and Seg‐1 were selected for the design of pan‐reactive real‐time RT‐PCR assays. The resulting mean Ct values for most of the strains tested in the Seg‐9 assay were slightly better (lower) when compared to those obtained in the Seg‐1 assay, indicating that these tests detect viral RNA with slightly different efficiencies, hence Seg‐1‐based assay was not reported here.

In contrast, the typing assays were designed targeting Seg‐2, the most variable and type‐specific region of the virus genome. A single set containing two primers and a probe was designed for the Seg‐9 pan‐reactive assay and for each of the Seg‐2 type‐specific assays. Previously published EHDV real‐time RT‐PCR assays were either designed from limited sequence data or use several primer pairs and/or probes (Clavijo et al., 2010; Viarouge et al., 2015; Wilson et al., 2015). The Seg‐2 nt sequence from the novel EHDV serotype (EHDV‐9), isolated from an Alpaca from Montagu district in the western Cape, South Africa, shows 69.7% nt identity to EHDV‐2 (AUS1979/05 – Accession no. AM744988) (Wright, 2013). Previously published pan‐reactive or type‐specific EHDV assays do not cover this novel EHDV serotype.

The group reactive assay targeting Seg‐9 is highly sensitive and specific to EHDV. All of the reference or field strains that were tested were detected and no amplification was generated with RNA from other related orbiviruses. All of the serotype‐specific assays also showed good results in terms of their sensitivity and specificity. There was no evidence of cross‐amplification with RNA templates from heterologous EHDV serotypes, by any of the type‐specific assays.

The assays detected a wide range of reference and field strains of EHDV from cell culture supernatant, diagnostic blood and tissue samples, indicating their diagnostic sensitivity and specificity and validity for molecular epidemiology studies of EHDV. The samples, tested in triplicate in different runs, showed a good reproducibility for simultaneous detection of IPC (the Beta‐actin gene), helping to assess the quality of test samples. The reaction conditions for the pan‐reactive and type‐specific EHDV assays were optimized to maximize sensitivity, using the same cycling conditions, for ease of performance of multiple assays in parallel.

The pan‐reactive (Seg‐9) and type‐specific (Seg‐2) assays can detect as few as to 2 copies of viral dsRNA per reaction, indicating that these assays would be able to detect the presence of EHDV in a diagnostic blood sample, even if the animal has only a low level of viraemia or has already developed neutralizing antibodies.

As previously demonstrated for BTV, type‐specific real‐time RT‐PCR assays are the most sensitive and reliable methods (that are currently available) for typing orbiviruses (Hoffmann et al., 2009; LeBlanc et al., 2010; Yin et al., 2010). The diagnostic specificity of the complete panel of EHDV typing assays described here relates primarily to their inability to detect Seg‐2 of non‐homologous types, while still detecting all available isolates from the homologous EHDV type. However, the relatively small number of EHDV isolates that are available, especially for serotypes 4, 5, 8 and 9, implies need for the wider validation.

As already seen with comparable assays for BTV, it is likely that novel EHDV strains will be identified that contain sequence changes in the primer or probe footprints used in these assays. It will, therefore, be necessary to redesign and refine the assay components to maintain their efficiency and efficacy (Maan et al., 2012). In some EHDV serotypes (−1, 2, 6 and 7), clear eastern and western topotypes have been identified, which can be differentiated using either the eastern or western topotype‐specific assays. It is possible that isolates belonging to different topotypes will also be identified for other EHDV serotypes, again requiring further assay development.

The assays described here are currently being used for diagnostic and/or research purposes, by the reference laboratories and research groups at The Pirbright Institute. In endemic areas, BTV and EHD viruses are often reported to co‐circulate, a situation that requires efficient and reliable diagnostic tool to positively identify and distinguish each of the causative agents. The group (pan‐reactive) and type‐specific assays described here were used to test and type all the EHDV isolates that are available in ORC at the Pirbright Institute (Mertens and Attoui, 2015), including the novel reassortant strain from USA containing RNA segments derived from EHDV‐6 and EHDV‐2 (Allison et al., 2010). In conclusion, the development of these assays provides a complete set of tools for the rapid, sensitive and specific detection and typing of EHDV that will help to implement appropriate surveillance and control measures.

## Supporting information


**Table S1.** (a) Analytical sensitivity of Seg‐9 pan‐reactive RT‐PCR assay with serially diluted dsRNA in RNA extracted from EHDV negative blood. (b) Limit of detection of Seg‐9 pan‐reactive RT‐PCR assay with serially diluted recombinant plasmid DNA.

**Table S2.** Analytical sensitivity and efficiency of type‐specific (Seg‐2) assays with serially diluted dsRNA standards.
