# Supplementary material for: Development of Real‐Time RT‐PCR Assays for Detection and Typing of Epizootic Haemorrhagic Disease Virus
Source: Transbound Emerg Dis. 2016 Feb 17;64(4):1120–32. doi: 10.1111/tbed.12477 (PMC5516135; doi:10.1111/tbed.12477)
Supplement: Supplementary file 1 — Table S1. (a) Analytical sensitivity of Seg‐9 pan‐reactive RT‐PCR assay with serially diluted dsRNA in RNA extracted from EHDV negative blood. (b) Limit of detection of Seg‐9 pan‐reactive RT‐PCR assay with serially diluted recombinant plasmid DNA. [file TBED-64-1120-s001.docx]

**Supplementary data**

Table S1a: Analytical sensitivity of Seg-9 group-specific RT-PCR assay with serially diluted dsRNA in RNA extracted from EHDV negative blood.

| **EHDV isolate serotype /designation/dilution** | **Mean Ct value for**  **EHDV-1e (AUS1995/02)** |
| --- | --- |
| EHDV-1e/AUS1995/02 undiluted | 18.3 |
| EHDV-1e/AUS1995/02/10^-0^ | 21.1 |
| EHDV-1e/AUS1995/02/10^-1^ | 24.3 |
| EHDV-1e/AUS1995/02/10^-2^ | 27.6 |
| EHDV-1e/AUS1995/02/10^-3^ | 30.5 |
| EHDV-1e/AUS1995/02/10^-4^ | 33.2 |
| EHDV-1e/AUS1995/02/10^-5^ | 38.5 |
| EHDV-1e/AUS1995/02/10^-6^ | 41.0 |
|  |  |
