# Supplementary material for: Development of Real‐Time RT‐PCR Assays for Detection and Typing of Epizootic Haemorrhagic Disease Virus
Source: Transbound Emerg Dis. 2016 Feb 17;64(4):1120–32. doi: 10.1111/tbed.12477 (PMC5516135; doi:10.1111/tbed.12477)
Supplement: Supplementary file 2 [file TBED-64-1120-s002.docx]

**Supplementary data**

Table S1b: Limit of detection of Seg-9 group-specific RT-PCR assay with serially diluted recombinant plasmid DNA.

| **Recombinant plasmid designation** | **Mean Ct value for**  **EHDV-1e (AUS1995/02), EHDV-2e (JAP1959/01), and EHDV-1w (NIG1967/01)** | **Number of copies/PCR** |
| --- | --- | --- |
| pEHDV 10^-6^ | 20.26 | 1.17x10^5^ |
| pEHDV 10^-7^ | 23.55 | 1.17x10^4^ |
| pEHDV 10^-8^ | 27.23 | 1.17x10^3^ |
| pEHDV 10^-9^ | 30.31 | 117 |
| pEHDV 10^-10^ | 33.50 | 11.7 |
| pEHDV 10^-10^ D1/2 | 35.10 | 5.8 |
| pEHDV 10^-10^ D1/4 | 36.02 | 2.9 |
| pEHDV 10^-10^ D1/8 | - | 1.5 |
| pEHDV 10^-11^ | - | 1.1 |
|  |  |  |

The limit of detection of EHDV Seg-9 assay is 3 copies of plasmid/PCR
