# Supplementary material for: Development of Real‐Time RT‐PCR Assays for Detection and Typing of Epizootic Haemorrhagic Disease Virus
Source: Transbound Emerg Dis. 2016 Feb 17;64(4):1120–32. doi: 10.1111/tbed.12477 (PMC5516135; doi:10.1111/tbed.12477)
Supplement: Supplementary file 3 — Table S2. Analytical sensitivity and efficiency of type‐specific (Seg‐2) assays with serially diluted dsRNA standards. [file TBED-64-1120-s003.docx]

**Supplementary data**

Table S2: Analytical sensitivity and efficiency of type-specific (Seg-2) assays with serially diluted dsRNA standards.

| **Virus isolate serotype/eastern or western** | **Virus isolate designation** | **Number of molecules detected** | **Efficiency** | **RSq** | **Slope** |
| --- | --- | --- | --- | --- | --- |
| **EHDV-1e** | AUS1995/02 | 3 | 100.5 | 0.995 | -3.3 |
| **EHDV-1w** | USA1955/01 | 4 | 88.1% | 1.00 | -3.64 |
| **EHDV-2e** | AUS1979/05 | 2 | 110.8 | 0.989 | -3.1 |
| **EHDV-2w** | CAN1962/01 | 6 | 98.9 | 0.995 | -3.34 |
| **EHDV-4w** | NIG1968/01 | 14 | 103.0% | 0.994 | -3.253 |
| **EHDV-5e** | AUS1977/01 | 128 | 104.4 | 0.997 | -3.2 |
| **EHDV-6e** | AUS1981/07 | 63 | 91.5 | 1.00 | -3.5 |
| **EHDV-6w** | BAR1983/01 | 5 | 94.8 | 1.00 | -3.5 |
| **EHDV-7e** | AUS1981/06 | 6 | 98.4 | 0.997 | -3.4 |
| **EHDV-7w** | ISR2006/013 | 139 | 96.8 | 1.00 | -3.4 |
| **EHDV-8e** | AUS1982/06 | 58 | 85.6 | 0.999 | -3.7 |
